# Emerging of Multidrug-Resistant Cronobacter sakazakii Isolated from Infant Supplementary Food in China

**DOI:** 10.1128/spectrum.01197-22

**Published:** 2022-09-29

**Authors:** Xin Gan, Menghan Li, Jin Xu, Shaofei Yan, Wei Wang, Fengqin Li

**Affiliations:** a Key Laboratory of Food Safety Risk Assessment, National Health Commission, China National Center for Food Safety Risk Assessment, Beijing, People’s Republic of China; Health Canada

**Keywords:** *Cronobacter sakazakii*, multidrug resistance, resistant gene, genome sequencing, large-scale surveillance

## Abstract

*Cronobacter* is a foodborne pathogen associated with severe infections in restricted populations and particularly with high mortality in neonates and infants. The prevalence and antimicrobial resistance (AMR) phenotype of *Cronobacter* cultured from powdered infant formula and supplementary food were studied. The virulence factors, AMR genes, and genomic environments of the multidrug-resistant isolates were further studied. A total of 1,055 *Cronobacter* isolates were recovered from 12,105 samples of powdered infant formula and supplementary food collected from 29 provinces between 2018 and 2019 in China. Among these, 1,048 isolates were from infant supplementary food and 7 were from powdered infant formula. Regarding antimicrobial resistance susceptibility, 11 (1.0%) isolates were resistant and two showed resistance to four antimicrobials (ampicillin [AMP], tetracycline [TET], sulfamethoxazole-trimethoprim [SXT], and chloramphenicol [CHL]), defined as MDR. These two MDR isolates were subsequently identified as Cronobacter sakazakii sequence type 4 (ST4) (C. sakazakii Crono-589) and ST40 (C. sakazakii Crono-684). Both MDR isolates contain 11 types of virulence genes and 7 AMR genes on their genomes. Meanwhile, the IncFIB plasmids of both MDR C. sakazakii isolates also harbored 2 types of virulence genes. Results of the genomic comparative analysis indicated that food-associated C. sakazakii could acquire antimicrobial resistance determinants through horizontal gene transfer (HGT).

**IMPORTANCE** As a foodborne pathogen, *Cronobacter* can cause serious infections in restricted populations and lead to death or chronic sequelae. Although a number of investigations showed that *Cronobacter* isolates are susceptible to most antimicrobial agents, MDR *Cronobacter* isolates, isolated mainly from clinical cases but occasionally from foods, have been reported in recent years. In this study, we successfully identified two MDR Cronobacter sakazakii isolates from infant foods based on nationwide surveillance and genome sequencing in China. Genomic analysis revealed that these two MDR C. sakazakii strains acquired resistance genes from other species via different evolution and transmission routes. It is important to monitor MDR C. sakazakii isolates in infant foods, and appropriate control measures should be taken to reduce the contamination with and transmission of this MDR bacterium.

## INTRODUCTION

*Cronobacter* is a Gram-negative, facultatively anaerobic, rod-shaped, non-spore-forming opportunistic pathogen that can be fatal to neonates and immunocompromised infants (1). This organism has been recovered from various sources, including beverages, vegetables, meat, soil, and dust. The genus *Cronobacter* consists of seven species, including Cronobacter sakazakii, C. dublinensis, C. turicensis, C. malonaticus, C. muytjensii, C. condimenti, and C. universalis (2–5). Among these seven species, C. sakazakii is the most predominant and associated with neonatal infections, whereas *C. malonaticus* is associated with adult infections (6–8). All age groups can be infected by this bacterium; however, newborns and the elderly are at the highest risk for infection (7). The major clinical symptoms caused by *Cronobacter* in infants include meningitis, bacteremia, and necrotizing enterocolitis, with mortality ranging from 40.0% to 80.0% (9, 10).

The contamination of powdered infant formula by *Cronobacter* constitutes a potential risk to human beings, especially infants. The prevalence of *Cronobacter* in samples of powdered infant formula (PIF) collected from Chinese retail markets between 2015 and 2017 was 2.8% (56/2,020) (11). Although PIF manufacturers have continuously strengthened the supervision of production processes, *Cronobacter* contamination still occurs constantly, which might be due to its wide distribution in environments like soil, water, and dust (12).

According to published data, key aspects of the pathogenesis and development of the disease caused by *Cronobacter* include the presence of virulence factors and antimicrobial resistance (AMR) genes. To date, virulence factors identified in *Cronobacter* include outer membrane proteins, efflux systems, iron acquisition systems, hemolysins, and so on (13). Moreover, the occurrence of AMR genes, especially those mediated by mobile genetic elements, in *Cronobacter* is another concern that limits the infectious therapeutic options for clinicians and others (14). Antimicrobial therapy is a common treatment of clinical infection caused by *Cronobacter* (15–18), but the occurrence of AMR genes, especially mediated by mobile genetic elements, in this bacterium limits the therapeutic options for clinicians (14). In particular, it is worth noting that MDR and even *mcr-1*- and *mcr-9*-carrying *Cronobacter* isolates have been detected in food, clinical, and animal specimens and in samples from a dead infant fed *Cronobacter*-contaminated PIF (19–22). Therefore, the emergence of antimicrobial resistance, particularly MDR, in *Cronobacter* in infant foods has become a potential public health concern. Few data on the prevalence of MDR *Cronobacter* in infant foods were available in China until whole-genome sequencing (WGS) began to be widely used as a powerful tool for characterization of virulence factors and AMR genes harbored by this bacterium. Here, we report the emergence of two MDR C. sakazakii isolates obtained in a nationwide foodborne-pathogen surveillance program between 2018 and 2019 in China. The genomic features of these two isolates and related pathogenic factors are characterized.

## RESULTS

### Prevalence of *Cronobacter* contamination in PIF and supplementary foods.

In total, 1,055 *Cronobacter* isolates were recovered from 12,105 (8.7%; 1,055/12,105) PIF and supplementary rice-based food samples collected from 29 provinces in China (see Table S1 in the supplemental material). Regarding the prevalence of *Cronobacter* contamination by year, 354 *Cronobacter* isolates were cultured from 8,105 (4.4%; 354/8,105) samples collected in 2018 and 701 from 4,000 (17.5%; 701/4,000) samples in 2019. In terms of the prevalence of *Cronobacter* contamination by food category, 7 isolates were cultured from 4,050 (0.2%; 7/4,050) PIF samples and 1,048 isolates from 8,055 (13.0%; 1,048/8,055) infant supplementary food samples. Based on the data described above, the contamination of *Cronobacter* in supplementary rice-based food samples was much higher than that in PIF (*P* < 0.01).

The prevalence of *Cronobacter* contamination among sampling locations was different (Table S1). In 2018, nine provinces had a prevalence of *Cronobacter* contamination higher than 10.0% (ranging from 23.0% to 13.3%) in supplementary foods, including Yunnan, Shanxi, Jiangsu, Guangxi, Gansu, Hubei, Shaanxi, Jiangxi, and Sichuan. In 2019, eight provinces had contamination rates higher than 20.0% (ranging from 27.0% to 22.5%), including Zhejiang, Hubei, Hunan, Shandong, Shaanxi, Sichuan, Shanxi, and Tianjin. It can be seen that the prevalence of *Cronobacter* contamination in samples of infant supplementary foods collected in 2019 was higher than in 2018.

### Antimicrobial susceptibility testing (AST) of *Cronobacter*.

Most *Cronobacter* isolates (92.3%; 974/1,055) were sensitive to all 12 antimicrobials tested, while 11 isolates (1.0%; 11/1,055) were resistant to at least one antimicrobial. These antimicrobial-resistant isolates were all from supplementary foods (Table 1). Among these 11 AMR isolates, 9 were resistant to a single antimicrobial (6 were resistant to chloramphenicol [CHL], and 1 each was resistant to ampicillin [AMP], nalidixic acid [NAL], and tetracycline [TET]). Furthermore, two isolates, designated Crono-589 and Crono-684, were MDR, with the resistance profile AMP-TET-SXT (sulfamethoxazole-trimethoprim)-CHL.

**TABLE 1:** Antimicrobial susceptibility of 1,055 *Cronobacter* isolates to 12 tested antimicrobial agents

Antimicrobial	No. (%) of isolates		
	Resistant	Intermediate	Susceptible
AMP	3 (0.3)	1 (0.1)	1,051 (99.6)
SAM	0 (0)	1 (0.1)	1,054 (99.9)
CTX	0 (0)	0 (0)	1,055 (100)
CAZ	0 (0)	0 (0)	1,055 (100)
IMP	0 (0)	0 (0)	1,055 (100)
NAL	1 (0.1)	0 (0)	1,054 (99.9)
CIP	0 (0)	0 (0)	1,055 (100)
TET	3 (0.3)	1 (0.1)	1,051 (99.6)
GEN	0 (0)	0 (0)	1,055 (100)
KAN	0 (0)	0 (0)	1,055 (100)
SXT	2 (0.2)	0 (0)	1,053 (99.8)
CHL	8 (0.8)	68 (6.5)	979 (92.8)

### Genomic characterization of two MDR strains.

As shown in Table 2, long-read sequencing of *Cronobacter* Crono-589 and Crono-684 successfully facilitated the construction of the complete genome sequence, including their chromosomes and plasmids. Both strains were identified as C. sakazakii. The total size of the chromosome in *Cronobacter* Crono-589 was 4,299,463 bp, with an average GC content of 56.9%, including 3,940 open reading frames (ORFs) and 109 RNAs. Two circular plasmids, identified as pCrono589-1 and pCrono589-2, were found in Crono-589. The total size of pCrono589-1 was ~158.3 kb, with an average GC content of 56.6%, including 148 ORFs, while the total size of pCrono589-2 was ~34.3 kb, with an average GC content of 47.4%, including 44 ORFs. In Crono-684, the total size of the chromosome was 4,364,183 bp, with an average GC content of 56.9%, including 3, 974 ORFs and 106 RNAs. Two circular plasmids, identified as pCrono684-1 and pCrono684-2, were also found in *Cronobacter* Crono-684. The total size of pCrono684-1 was ~118.1 kb, with an average GC content of 57.2%, including 111 ORFs, while the total size of pCrono684-2 was ~94.3 kb, with an average GC content of 49.6%, including 121 ORFs.

**TABLE 2:** Features of the genomes and plasmids identified in two MDR C. sakazakii isolates

Strain	Location	Size (bp)	G+C content (%)	No. of ORFs	No. of RNAs	ST or Inc type	Virulence genes	AMR genes	AMR spectrum
Crono-589	Chromosome	4,299,463	56.9	3,940	109	40	*cusC*, *fkpA*, *flhA*, *hfq*, *hha*, *higB*, *higB1*, *higB2*, *hlyIII*, *nanA*, *nanK*, *nanT*, *ompA*, *ompX*, *vgrG1*, *zpx*	*bla*_CSA-1_, *CRP*, *EF-Tu*, *emrB*, *GlpT*, *H-NS*, *msbA*	AMP-TET-SXT-CHL
	Plasmid pCrono589-1	158,317	56.6	148		IncFIB	*cpa*, *iucA*, *iucB*, *iucC*, *iucD*	*aadA2*, *bla*_TEM-1_, *catII*, *dfrA12*, *LAP-2*, *mphA*, *qnrS1*, *sul1*, *tet*(A)	
	Plasmid pCrono589-2	34,303	47.4	44		IncFIB			

Crono-684	Chromosome	4,364,183	56.9	3,974	106	4	*cusC*, *fkpA*, *flhA*, *hfq*, *hha*, *higB*, *higB2*, *hlyIII*, *nanA*, *nanK*, *nanT*, *ompA*, *ompX*, *vgrG1*, *zpx*	*bla*_CSA-2_, *CRP*, *EF-Tu*, *emrB*, *GlpT*, *H-NS*, *msbA*	AMP-TET-SXT-CHL
	Plasmid pCrono684-1	118,144	57.2	111		IncFIB	*cpa*, *iucA*, *iucB*, *iucC*, *iucD*		
	Plasmid pCrono684-2	94,206	49.6	121		IncY		*bla*_TEM-135_, *dfrA14*, *floR*, *qnrS1*, *tet*(A)	

### Subtyping of the MDR strains.

Crono-589 and Crono-684 were further subtyped as sequence type 40 (ST40) (clonal complex CC40) and ST4 (CC4), respectively, after submission of their FASTA files to the PubMLST *Cronobacter* database. Crono-589 carried two IncFIB plasmids, while Crono-684 carried IncFIB and IncY plasmids.

### *In silico* virulence gene identification.

Combined, the MDR strains harbored 13 types and 21 virulence genes (Table 2). Most of these genes were located on chromosome, including genes encoding zinc-containing metalloprotease (*zpx*), RNA-binding protein (*hfq*), outer membrane proteins (*ompA* and *ompX*), sialic acid utilization (*nanAKT*), macrophage infectivity potentiator (*fkpA*), hemolysin III (*hlyIII*), an IbeB homolog (*cusC*), expression-modulating protein (*hha*), valine-glycine repeat G family protein (*vgrG1*), members of the RelE/YieB family of bacterial toxin-antitoxins (*higB* and *higB2*), and a component of the flagellum export apparatus (*flhA*). Crono-589 carried another *higB* variant, *higB1*. The plasminogen activator (*cpa*) and iron acquisition system (*iucABCD*) genes were also found to be located on the IncFIB plasmids of both isolates.

### *In silico* identification of AMR genes.

Both MDR isolates carried 7 AMR genes on their chromosomes (Table 2). Among them, *msbA*, *GlpT*, *EF-Tu*, *emrB*, *bla*_CSA-1_, and *bla*_CSA-2_ can mediate resistance to nitroimidazole, fosfomycin, elfamycin, fluoroquinolone, and cephalosporin resistance, respectively. The *CRP* gene confers resistance to macrolides, fluoroquinolones, and penams, while the *H-NS* gene can lead to macrolide, fluoroquinolone, penam, cephalosporin, cephamycin, and tetracycline resistance. Meanwhile, plasmid pCrono589-1 was found to harbor 9 AMR genes: *mphA*, *LAP-2*, *bla*_TEM-1_, *sul1*, *dfrA12*, *catII*, *aadA2*, *tet*(A), and *qnrS1*. pCrono684-2 was found to harbor 5 AMR genes: *drfA14*, *floR*, *bla*_TEM-135_, *qnrS1*, and *tet*(A). Among them, *tet*(A), *mphA*, *aadA2*, *qnrS1*, and *sul1* can mediate tetracycline, macrolide, aminoglycoside, fluoroquinolone, and sulfonamide resistance, respectively. In addition, *catII* and *floR* can mediate resistance to phenicols, and *dfrA12* and *dfrA14* can mediate resistance to diaminopyrimidine. Three genes can mediate multiantimicrobial resistance, including the β-lactamase genes *bla*_TEM-1_ and *bla*_TEM-135_, which can mediate resistance to monobactams, cephalosporins, penams, and penems, and *LAP-2*, which can mediate resistance to fluoroquinolones, aminoglycosides, tetracycline and rifamycin. Moreover, no AMR genes were identified in pCrono589-2 and pCrono684-1.

Since rates of CHL-resistant (0.8%, 8/1,055) and -intermediate (6.5%, 68/1055) phenotypes were slightly higher than those for other antimicrobials, we further evaluated the presence of CHL resistance genes, including *catII* and *floR*, in all CHL nonsusceptible isolates by PCR. A total of 35 isolates (46.1%, 35/76), including 5 CHL-resistant isolates and 30 CHL-intermediate isolates, were positive for either *catII* or *floR* or both. Among these, 4.0% (3/76) carried both *catII* and *floR*, 32.9% (25/76) carried the *catII* gene alone, and 9.2% (7/76) carried the *floR* gene alone. Of the 3 isolates positive for both *catII* and *floR*, one was CHL resistant and the other 2 were intermediate. In addition, 41 phenotypically CHL-nonsusceptible isolates were negative for *catII* and *floR*; this implied that a mechanism for resistance to CHL other than AMR genes might exist.

### Genomic analysis of plasmids.

**FIG 1:**
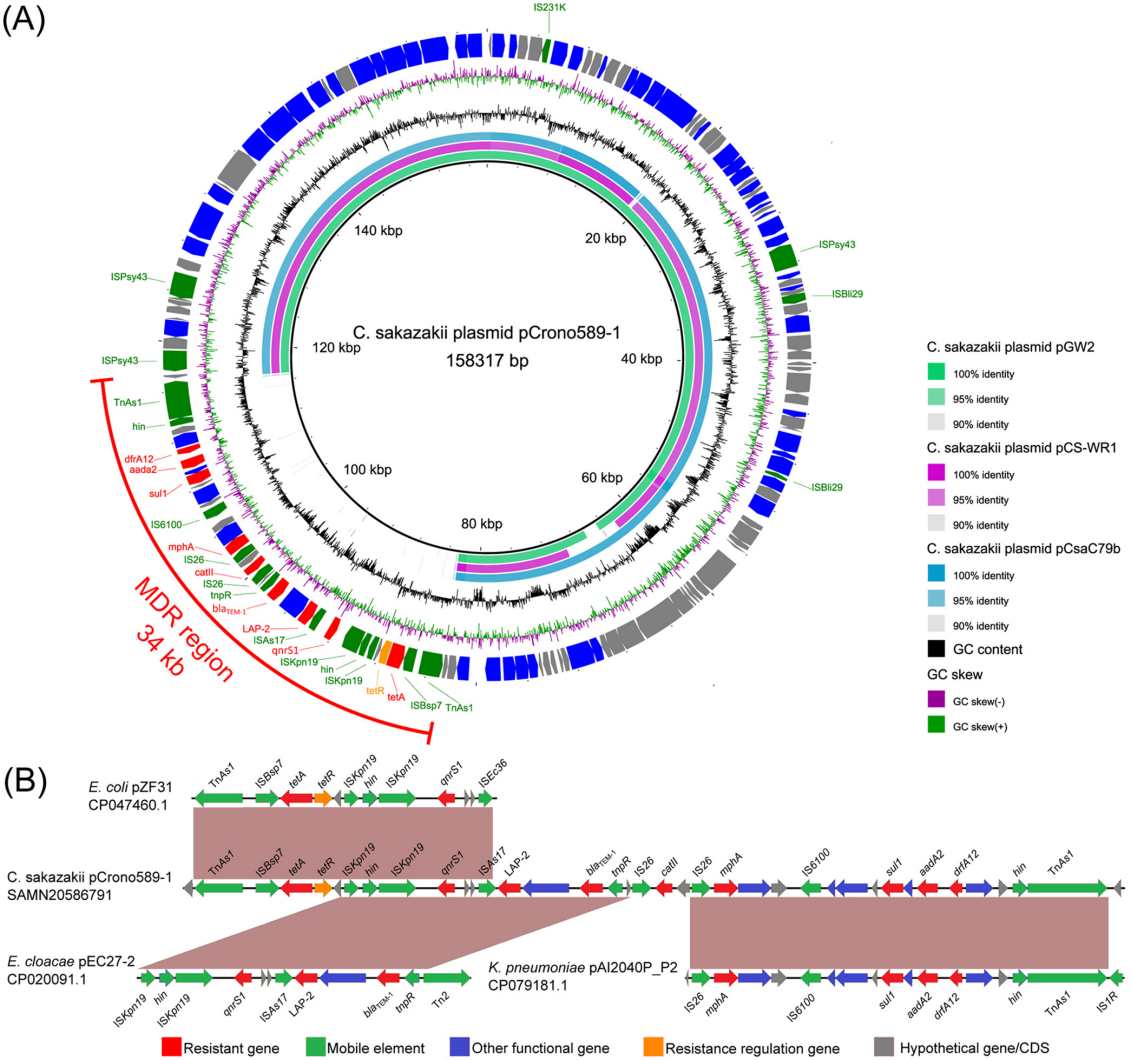
(A) Circular schematic map of pCrono589-1 plasmid in comparison to those in three C. sakazakii isolates used in this study. Coding sequences (CDSs) are shown as arrows. Antibiotic resistance genes are highlighted in red. Mobile gene elements are highlighted in green. Other functional genes are highlighted in blue. Antibiotic resistance regulation genes are highlighted in orange. Hypothetical proteins are highlighted in gray. Two rings represent GC skew [(G − C)/(G + C)] and GC content. The three circles represent C. sakazakii plasmids pGW2, pCS-WR1, and pCsaC79b. (B) Linear sequence comparison of the pCrono589-1 MDR region with three plasmids. Brown shading represents regions of homology. CDSs are shown as arrows. Antibiotic resistance genes are highlighted in red. Mobile gene elements are highlighted in green. Other functional genes are highlighted in blue. Antibiotic resistance regulation genes are highlighted in orange. Hypothetical proteins are highlighted in gray.

Linear sequence comparison was performed using plasmid pCrono589-1 along with three C. sakazakii plasmids, including NMI5563_17 plasmid pCS-WR1 (MT759836.1), C79 plasmid pCsaC79b (CP049145.1), and GZcsf-1 plasmid pGW2 (CP028976.1), as the closest common hits of online BLAST results against the NCBI nt/nr database. The query cover values for pCrono589-1 with these reference plasmids were from 73.0% to 78.0%, and identity was >97.95%, which clearly displayed most of the ortholog regions (Fig. 1A). Notably, these four plasmids shared a similar overall genetic backbone, but only pCrono589-1 carried an MDR region (34 kb). This 34-kb MDR region was encoded by two copies of transposons (Tn*As1*) in the opposite direction with AMR genes flanked by insertion sequences. Downstream of Tn*As1*, the tetracycline resistance genes *tet*(A) and *tet*(R) were flanked by IS*Bsp7* upstream and IS*Kpn19* downstream to them, followed by the DNA invertase gene *hin*. Next to *hin*, the plasmid-mediated quinolone resistance (PMQR) gene *qnrS1* was found to be flanked by IS*Kpn19*, followed by the IS*As17*-mediated Ambler class A beta-lactamase gene *LAP-2*, conferring resistance to quinolones. Downstream of this, another beta-lactamase gene, *bla*_TEM_, was found on the transposon Tn*pR*, followed by the macrolide resistance gene *mphA* with one insertion sequence (IS*26*) upstream of it. Finally, the *sul1*, *aadA2*, and *dfrA12* genes, conferring resistance to sulfonamides, aminoglycosides, and diaminopyrimidines, respectively, were flanked by IS*6100* and *hin*. In addition to AMR genes, there was a *qacE*Δ*1* gene, which mediates tolerance to acridine dye, disinfecting agents, and intercalating dyes, located between *sul1* and *aadA2*. Comparison results showed high similarity (identity > 99%) to those in plasmids from Escherichia coli (pZF31; CP047460.1), Enterobacter cloacae (pEC27-2; CP020091.1), and Klebsiella pneumoniae (pAI2040P_P2; CP079191.1) (Fig. 1B).

**FIG 2:**
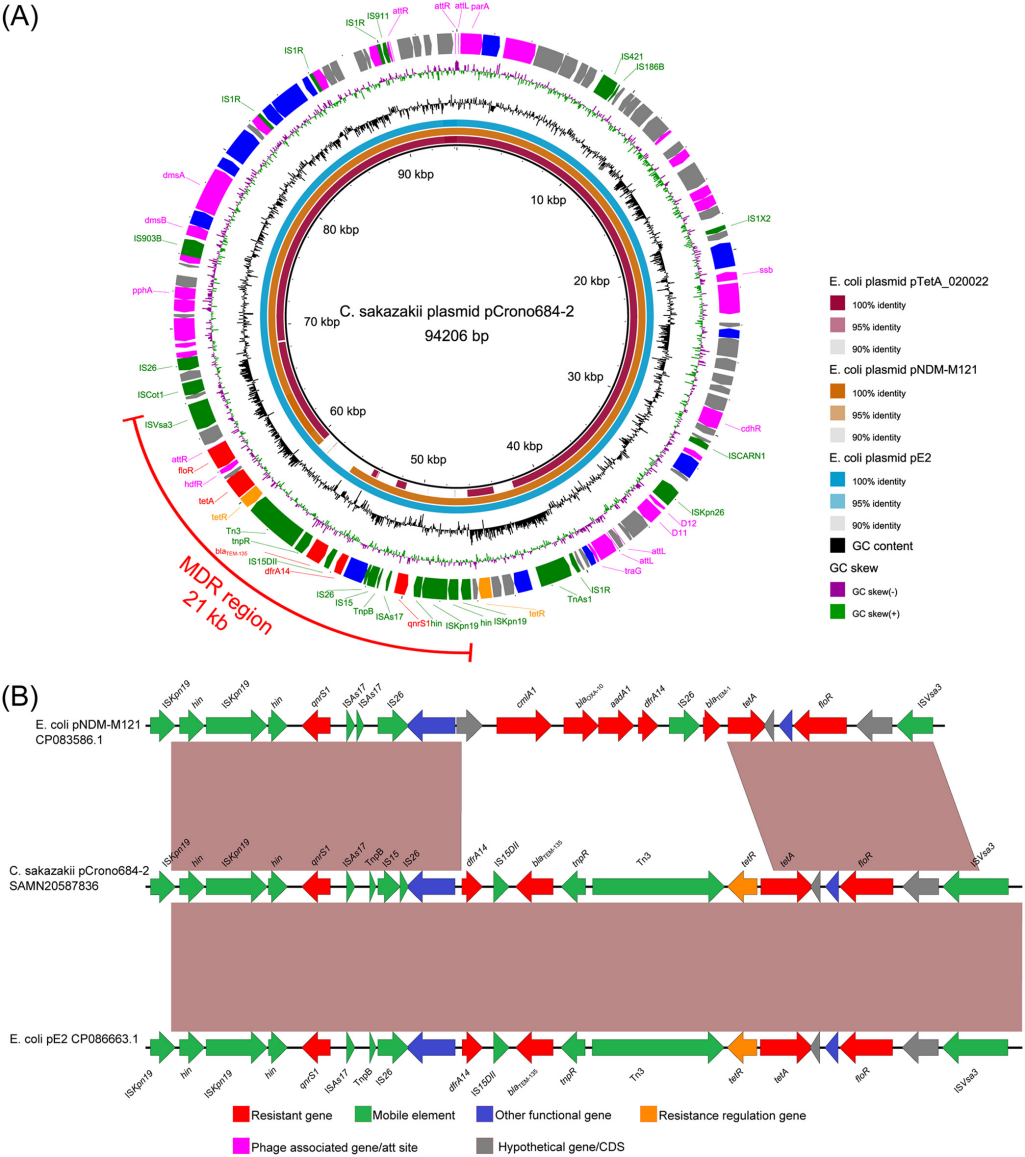
(A) Circular schematic map of pCrono684-2 plasmid in comparison to those in three E. coli isolates used in this study. CDSs are shown as arrows. Antibiotic resistance genes are highlighted in red. Mobile gene elements are highlighted in green. Other functional genes are highlighted in blue. Antibiotic resistance regulation genes are highlighted in orange. Phage-associated genes are highlighted in purple. Hypothetical proteins are highlighted in gray. Two rings represent GC skew [(G − C)/(G + C)] and GC content. The three circles are the E. coli plasmids pTetA_020022, pNDM-M121, and pE2. (B) Linear sequence comparison of pCrono684-2 MDR region with two plasmids. Brown shading represents regions of homology. CDSs are shown as arrows. Antibiotic resistance genes are highlighted in red. Mobile gene elements were highlighted in green. Other functional genes are highlighted in blue. Antibiotic resistance regulation genes are highlighted in orange. Hypothetical proteins are highlighted in gray.

Interestingly, the plasmid pCrono684-2 showed high similarity with three plasmids from E. coli, including pTetA_020022 (CP032890.1), pNDM-M121 (CP083586.1), and pE2 (CP086663.1). The query cover values for pCrono684-2 with three reference plasmids were from 85.0% to 100.0% and the identity was >99.95% (Fig. 2A). According to the phage annotation results by PHASTER, these four plasmids were phage-like plasmids and contained an intact prophage similar to phage Escher RCS47 (NCBI Reference Sequence: NC_042128.1), which covered 99.84% of the plasmid pCrono684-2. Furthermore, pCrono684-2 has complete phage-building proteins, accompanied by multiple phage *att* sites (Fig. 2A). In addition, pCrono684-2 also carried a 21-kb MDR region, showing high identity (>99%) with pE2 (CP086663.1) and partial similarity with pNDM-M121 (CP083586.1) (Fig. 2B). In the 21-kb MDR region located on pCrono684-2, two copies of insertion sequences (IS*Kpn19*) and two copies of DNA invertase genes (*hin*) were found upstream of the *qnrS1* gene, followed by three insertion sequences (IS*As17*, IS*15*, and IS*26*) and one transposon (Tn*pB*). Next to this, the diaminopyrimidine resistance gene *dfrA14* followed by the beta-lactamase gene *bla*_TEM-135_ was found to be flanked by one insertion sequence (IS*15DII*) and transposon (Tn*pR*). Finally, the tetracycline resistance genes [*tet*(R) and *tet*(A)] and phenicol resistance gene (*floR*) were found to be flanked by one transposon (Tn*3*) and insertion sequence (IS*Vsa3*).

## DISCUSSION

In the clinical setting, *Cronobacter* has been isolated from feces, sputum, blood, bone marrow, and cerebrospinal fluid, with newborns younger than 12 months and the elderly being the most susceptible populations (23, 24). To date, this bacterium has been found in a wide range of environments, including water, food, and soil (25). Through new identification techniques and genomic sequencing, we have learned a great deal about the bacteria that make up this genus (26, 27). In the current study, considering the risk of contamination of *Cronobacter* in infant food, we investigated the prevalence and AMR phenotype as well as genotype of this bacterium cultured from infant formula and supplementary food.

The total detection rate of *Cronobacter* in all PIF and supplementary food samples was 8.7%, similar to those reported in Brazil (8.6%; 13/152) (28). Of note, the *Cronobacter* contamination in cereal-based foods (prevalence up to 27.0%) was much more severe than that in PIF. This is consistent with that reported by Carvalho et al. (17.3%) (28). The consumption of supplementary foods is mainly for infants >6 months old, whose immune systems are not fully developed. However, there is no maximum tolerance limit for *Cronobacter* in related supplementary foods in China. The consumption of supplementary foods contaminated by *Cronobacter* would lead to infection in this population. Therefore, complicated strategies, including development of new safety criteria along with hygienic control measures during food production and risk communication with varied stakeholders, need to be adopted in order to improve the safety of infant food products.

Antimicrobial abuse in agricultural and clinical settings favored the development of resistance to different antibiotics in *Cronobacter* and then led to the spread of antimicrobial-resistant bacteria/genes in the environment (29–32). Although *Cronobacter* isolates are generally susceptible to the antimicrobial agents most often used clinically, resistance to one or more antimicrobials such as cephalothin and streptomycin (33, 34) and even β-lactams or colistin (19–21) has been frequently reported recently. Our previous study showed that the resistance rate of 417 *Cronobacter* isolates collected from 2012 to 2014 was 1.9% (35). No AMR isolates were found among 52 *Cronobacter* isolates, as reported by Holý et al. (36). Among the 11 resistant isolates obtained in the present study, resistance to CHL was detected most often (8/11), followed by resistance to AMP (3/11), TET (3/11), SXT (2/11), and NAL (1/11). In addition, 68 isolates (6.5%; 68/1,055) also showed intermediate resistance to CHL. In particular, a strain of *Cronobacter* with intermediate resistance to CHL could exhibit a stable resistance phenotype (37). Notably, 46.1% of the CHL-nonsusceptible strains even with the intermediate resistance phonotype were also found to carry genes (*catII* or *floR*) conferring resistance to this antibiotic, suggesting that resistance to CHL might be an emerging trend in foodborne *Cronobacter*. Traditionally, ampicillin, chloramphenicol, tetracycline, and trimethoprim-sulfamethoxazole, to which isolates showed resistance in our study, are used for clinical treatment of infection by *Enterobacteriaceae*, including *Cronobacter* (9, 30). Hence, AMR *Cronobacter* poses an important, complex, and priority public health challenge.

Although the prevalence of AMR *Cronobacter* isolates (1.0%; 11/1,055) was relatively low in this study, two MDR isolates with the resistance profile AMP-TET-SXT-CHL were found. Recently, several cases of neonatal infection with MDR *Cronobacter* that caused deaths or significantly impaired mental and physical development have been reported (20, 22, 38). Consumption of these food-related MDR *Cronobacter* isolates in the supplementary infant foods would lead to infections in infants with failure or limited choice of clinical therapy. Therefore, our finding highlights the importance of monitoring of MDR *Cronobacter* along the infant food production chain to address the risks associated with development, selection, and dissemination of foodborne resistant *Cronobacter* and resistance determinants.

According to our previous study, C. sakazakii is also the predominant species (70.8%; 208/294) cultured from infant foods (39, 40). C. sakazakii is the major species mainly related to neonatal infections (6). In this study, the two MDR C. sakazakii isolates belonged to ST4 and ST40. ST4 is a predominant sequence type in *Cronobacter* clinical isolates cultured from specimens of neonate and infant with symptoms of meningitis, bacteremia and necrotizing enterocolitis (NEC) (41, 42). This sequence type was also the predominant sequence type among *Cronobacter* isolates from PIF, while ST40 was commonly cultured from the environment, including multiple food processing facilities, and PIF, with only one isolate from a clinical case reported (25, 43–45). Up to May 2022, the *Cronobacter* database of PubMLST had 3,424 sequences deposited, belonging to 94 STs, among which 425 sequences were from clinical isolates, with ST4 as the most predominant (27.1%; 115/425) sequence type. Regarding the 65 ST40 isolates in PubMLST, 19 (29.2%; 19/65) were from the environment and 4 (6.2%; 4/65) from clinical samples. Therefore, these two MDR C. sakazakii isolates would have the potential to cause clinical infections once infants consumed food contaminated by them.

So far, C. sakazakii is known to cause septicemia, NEC, and meningitis. Hence, specific genes that are involved in infection attract more attention. In this study, 13 types including 21 virulence genes were identified from the genomes of both MDR C. sakazakii isolates. Of note, several virulence genes have been reported from C. sakazakii clinical isolates. The *hlyIII* gene, encoding hemolysin III, was found in C. sakazakii BAA-894, isolated from the neonatal intensive care unit outbreak in 2001 (46), while the proteins encoded by *zpx* and *cusC* genes, which invade human brain microvascular endothelial cells, can help bacteria cross the blood-brain barrier or cause extensive cellular destruction in neonates with NEC (47, 48), respectively. The virulence genes *ompA, ompX*, *nanA*, *nanK*, *nanT*, *hha*, *flhA*, and *hfq* are involved in enhancing invasion and adhesion of C. sakazakii (13, 49, 50). Furthermore, virulence genes located on the IncFIB plasmids in C. sakazakii were reported (14, 32). In this study, virulence genes, including *cpa* and *iucABCD*, which could support bacterial growth in human hosts and which were detected in isolates from clinical cases of C. sakazakii infection (51–54), were also found in the IncFIB plasmids of both MDR isolates’ genes. All the virulence genes detected in both MDR C. sakazakii isolates implied a potential infection risk for infants upon consuming food contaminated by these strains.

Regarding the *in silico* presence of chromosome-borne AMR genes, both MDR isolates had the same efflux genes (*msbA* and *emrB*), antibiotic efflux-modulating regulatory system genes (*CRP* and *H-NS*), and antibiotic target alteration genes (*GlpT* and *EF-Tu*) (28). Lepuschitz et al. (41) reported that of 21 C. sakazakii isolates, 12 carried *msbA*, *emrB*, *CRP*, *H-NS*, and *GlpT* genes. Recent studies showed that the above-mentioned AMR genes could be detected in all C. sakazakii and other *Enterobacteriaceae* bacteria, suggesting that these genes may be inherent AMR genes and vertically transmitted in C. sakazakii (13, 32). This aspect is particularly relevant in the context of increasing antibiotic resistance (55). For instance, fosfomycin is considered a useful antibiotic for treating MDR bacterial infections, but a *GlpT* gene encoding resistance to fosfomycin was also found in both MDR isolates in our study. In addition, these two MDR C. sakazakii isolates carry β-lactamase class C resistance genes (*bla*_CSA-1_ and *bla*_CSA-2_) and possess resistance to most first- and second-generation cephalosporins (32).

It is well known that resistance genes can be transmitted vertically and horizontally through mobile genetic elements (MGEs) like plasmids, integrons, transposons, and so on (56, 57). Both MDR C. sakazakii isolates had one plasmid with several AMR genes, including the TEM β-lactamase genes *bla*_TEM-1_ and *bla*_TEM-135_, the tetracycline resistance gene *tet*(A), the sulfonamide resistance gene *sul1*, the diaminopyrimidine resistance genes *dfrA12* and *dfrA14*, and the phenicol resistance genes *catII* and *floR*. All these could explain their AMR phenotype (AMP-TET-SXT-CHL). Other AMR genes (*mphA*, *qnrS1*, and *LAP-2*) were also identified in the plasmids. As far as we know, the *bla*_TEM_ gene-carrying plasmid in C. sakazakii is rarely reported from food isolates (20, 58). This is the first report on C. sakazakii isolates carrying *bla*_TEM_ plasmids cultured from infant food in China.

In addition to the ~34-kb MDR region, the genetic backbone of plasmid pCrono589-1 showed high similarity to other plasmids harbored by C. sakazakii, indicating that this plasmid could be stably transmitted among C. sakazakii isolates, whereas the MDR region could be partially found in plasmids in other *Enterobacteriaceae* species, such as pZF31 in *tet*(X4)-positive E. coli (CP047460.1) isolated from swine feces (59), pEC27-2 (CP020091.1) in a clinical isolate of E. cloacae that was resistant to carbapenem and colistin (60), and pAI2040P_P2 (CP079181.1) in a K. pneumoniae strain that was resistant to carbapenem and was obtained from urine samples from a urinary tract infection case. Moreover, those AMR genes were found to be flanked by several insertion sequences and transposons, which could mediate genetic exchange between or within species (61). The structurally similar mobile elements contained in these reported plasmids implied that the MDR region in plasmid pCrono589-1 is more likely acquired from resistant plasmids carried in other *Enterobacteriaceae* species than from the same species.

Unlike pCrono589-1, the genetic backbone of pCrono684-2 was not homologous to any *Cronobacter* plasmids but showed high similarity to three E. coli plasmids. In particular, pCrono684-2 was nearly identical to the E. coli E2 plasmids pE2 (CP086663.1) (99.97%) and pNDM-M121 (CP093586.1) (99.95%), indicating that this plasmid may be entirely acquired from E. coli. The resistant genes in plasmid pCrono684-2 were also found to be flanked by several insertion sequence or transposons, most of which were also found in E. coli plasmids, suggesting the potential transmission of plasmids between these species. Furthermore, PHASTER analysis indicated that Crono-684-p2 is a phage-like plasmid. Phage-like plasmids were not restricted to E. coli and could represent as a new vehicle to mediate the interspecies transmission of AMR genes (62). Overall, multiple mobile genetic elements around AMR genes, including insertion sequences, transposons, and other regulators, might contribute to the transmission of AMR genes or resistance plasmids between species. The above findings with regard to plasmids pCrono589-1 and pCrono684-2 revealed two different routes by which C. sakazakii could acquire resistance from other species, which would further help investigate the resistant mechanisms of such MDR C. sakazakii isolates.

### Conclusion.

In conclusion, our study revealed the prevalence of *Cronobacter* in infant foods across 29 provinces from 2018 to 2019 in China, with a relatively high detection rate in infant supplementary foods. Moreover, a total of 11 AMR *Cronobacter* isolates were identified, and two were MDR C. sakazakii isolates with the antimicrobial resistance profile AMP-TET-SXT-CHL. The sequence types of these two MDR C. sakazakii isolates were ST4 and ST40. The genomic analysis of MGEs in the plasmid-mediated MDR region of both C. sakazakii isolates revealed that isolates with similar antimicrobial resistance phenotypes may have obtained resistance genes from other species by different evolutionary and transmission routes. To the best of our knowledge, this is the first report of MDR C. sakazakii isolates carrying *bla*_TEM_ plasmids cultured from infant foods in China. Considering the possibility that these resistances emerging in infant food may be transferred to infants via the food chain, it is important to monitor the antimicrobial susceptibility and resistance mechanisms of MDR C. sakazakii isolates along the infant food supply chain.

## MATERIALS AND METHODS

### Bacterial isolation.

A total of 12,105 samples of powdered infant formula and cereal-based supplementary food were collected between 2018 and 2019 from 29 provinces in China. Among these samples, 8,105 (4,050 for PIF and 4,055 for cereal-based supplementary food) were from 2018 and 4,000 cereal-based supplementary food samples were from 2019. All samples were screened for contamination with *Cronobacter* using a modified method based on the China National Food Safety Standard (63). Briefly, a 100-g test portion sample was mixed with 900 mL buffered peptone water (BPW; Beijing Land Bridge Technology Ltd., Beijing, China) and incubated at 37°C for 18 h ± 2 h. Then, 1 mL of mixed pre-enrichment sample was transferred into a tube containing 10 mL modified lauryl sulfate tryptose broth (mLST)/vancomycin medium (Beijing Land Bridge Technology Ltd., Beijing, China) and incubated at 41.5°C for 24 h ± 2 h. A loopful of mLST/vancomycin (approximately 10 μL) culture was streaked on Brilliance Enterobacter sakazakii agar (DFI; Oxoid, England) and incubated at 41.5°C for 24 ± 2 h. Suspected colonies were selected, inoculated onto tryptone soy agar (TSA; Beijing Land Bridge Technology Ltd., Beijing, China) plates, and incubated at 37°C for 24 h. Colonies on TSA plates were further identified by both analysis on a Vitek 2 compact instrument (bioMérieux, France) and amplification of the internal transcribed spacer (ITS) as previously reported (64).

### Antimicrobial susceptibility testing.

All confirmed *Cronobacter* isolates were subjected to AST using Biofosun Gram-negative panels (Fosun Diagnostics, Shanghai, China) by the broth dilution method (65). The panel of 12 antimicrobial compounds includes the penicillin ampicillin (AMP; 0.5 to 64 μg/mL), the β-lactam–β-lactamase inhibitor combination ampicillin-sulbactam (SAM; 0.25 to 64/32 μg/mL), the cephems cefotaxime (CTX; 0.125 to 16 μg/mL) and ceftazidime (CAZ; 0.25 to 32 μg/mL), the carbapenem imipenem (IMP; 0.03 to 4 μg/mL), the aminoglycosides gentamicin (GEN; 0.5 to 64 μg/mL) and kanamycin (KAN; 0.5 to 64 μg/mL), tetracycline (TET; 0.5 to 64 μg/mL), the fluoroquinolone ciprofloxacin (CIP; 0.06 to 8 μg/mL), the quinolone nalidixic acid (NAL; 0.5 to 64 μg/mL), the folate pathway inhibitor sulfamethoxazole-trimethoprim (SXT; 0.125/2.375 to 16/304 μg/mL), and the phenicol chloramphenicol (CHL,2 to 256 μg/mL). Escherichia coli ATCC 25922 was used for quality control. All results were interpreted according to the Clinical and Laboratory Standards Institute (CLSI) interpretive standard M100, 29th edition.

### DNA extraction and whole-genome sequencing.

Two confirmed MDR *Cronobacter* isolates were inoculated onto TSA plates and incubated overnight at 37°C to obtain pure clones. Genomic DNA (gDNA) was extracted and purified using a TIANamp bacterial DNA kit (DP302-02; Tiangen Biotech Co., Ltd., China) according to the manufacturer’s instructions. Whole-genome sequencing was performed with a combination of the Illumina HiSeq 2000 (Illumina Inc., San Diego, CA, USA) and Pacific Biosciences Sequel II sequencing platforms (Pacific Biosciences, Menlo Park, CA, USA). The sequence data from the Illumina platform were used to proofread the PacBio assembly sequence. SMRT Analysis v2.3.0, Consed v28.0, and NCBI PGAP v4.8 were used for *de novo* assembly, generating complete and closed chromosome or plasmid sequences and automatically annotating them, respectively. Prokka v1.14.5 (66), KOALA v2.2 (https://www.kegg.jp/blastkoala/), and the PubMLST database (http://pubmlst.org/cronobacter/) were used to perform the genome annotation.

### Identification and *in silico* subtyping.

The species identification and multilocus sequence typing (MLST) of *Cronobacter* was performed *in silico* for both MDR isolates by uploading their whole-genome sequences (FASTA files) to the PubMLST *Cronobacter* database (http://pubmlst.org/cronobacter/). PlasmidFinder 2.1 was used to identify plasmid replicon types (Inc groups) (https://cge.food.dtu.dk/services/PlasmidFinder/) (67).

### Identification of virulence factors, AMR genes, and prophages.

Virulence genes were selected based on a careful literature review (13, 47–53, 68–72) and were screened among the studied genomes using the BLASTN algorithm (73) with 90% minimum nucleotide identity and 90% alignment length coverage. AMR genes were identified with the Comprehensive Antimicrobial Resistance Database (CARD) (74) with 90% identity and 95% query coverage as cutoffs. Prophage sequences within plasmids were identified and annotated by PHASTER (PHAge Search Tool Enhanced Release) (75).

### Genomic comparison and visualization of the genome.

Sequence comparisons were carried out with BLASTN and visualized using Easyfig v2.2.5 (76). A circular genome comparison graph was constructed with BRIG v0.95 (77).

### Data availability.

The nucleotide sequences of two MDR *Cronobacter* isolates have been deposited in GenBank under the accession numbers CP080591 (Crono-589 chromosome), CP080592 (pCrono589-1), CP080593 (pCrono589-2), CP080594 (Crono-684 chromosome), CP080595 (pCrono684-1), CP080596 (pCrono684-2), and BioProject PRJNA752220.
